# CDK9 inhibition strategy defines distinct sets of target genes

**DOI:** 10.1186/1756-0500-7-301

**Published:** 2014-05-16

**Authors:** Judit Garriga, Xavier Graña

**Affiliations:** 1 Fels Institute for Cancer Research and Molecular Biology, AHP bldg., room 308, 3307 North Broad St., Philadelphia, PA 19140, USA; 2 Department of Biochemistry, Temple University School of Medicine, 3307 North Broad St., Philadelphia, PA 19140, USA

**Keywords:** Transcription, CDK9, RNA polymerase II, CDKs, Control of gene expression

## Abstract

**Background:**

CDK9 is the catalytic subunit of the Positive Transcription Elongation Factor b (P-TEFb), which phosphorylates the CTD of RNAPII and negative elongation factors enabling for productive elongation after initiation. CDK9 associates with T-type cyclins and cyclin K and its activity is tightly regulated in cells at different levels. CDK9 is also the catalytic subunit of TAK (Tat activating Kinase), essential for HIV1 replication. Because of CDK9′s potential as a therapeutic target in AIDS, cancer, inflammation, and cardiomyophathy it is important to understand the consequences of CDK9 inhibition. A previous gene expression profiling study performed with human glioblastoma T98G cells in which CDK9 activity was inhibited either with a dominant negative mutant form of CDK9 (dnCDK9) or the pharmacological inhibitor Flavopiridol unveiled striking differences in gene expression effects. In the present report we extended these studies by (1) using both immortalized normal human fibroblasts and primary human astrocytes, (2) eliminating potential experimental variability due to transduction methodology and (3) also modulating CDK9 activity with siRNA.

**Findings:**

Striking differences in the effects on gene expression resulting from the strategy used to inhibit CDK9 activity (dnCDK9 or FVP) remain even when potential variability due to viral transduction is eliminated. siRNA mediated CDK9 knockdown in human fibroblasts and astrocytes efficiently reduced CDK9 expression and led to potent changes in gene expression that exhibit little correlation with the effects of dnCDK9 or FVP. Interestingly, *HEXIM1* a validated CDK9 target gene, was found to be potently downregulated by dnCDK9, FVP and siCDK9, but the cluster of genes with expression profiles similar to *HEXIM1* was small. Finally, cluster analysis of all treatments revealed higher correlation between treatments than cell type origin.

**Conclusion:**

The nature of the strategy used to inhibit CDK9 profoundly affects the patterns of gene expression resulting from CDK9 inhibition. These results suggest multiple variables that affect outcome, including kinetics of inhibition, potency, off-target effects, and selectivity issues. This is particularly important when considering CDK9 as a potential target for therapeutic intervention.

## Findings

CDK9 is the catalytic subunit of the positive Transcription Elongation Factor b (P-TEFb), which associates with T-type cyclins (T1 and T2) and cyclin K regulatory subunits (reviewed in [1-3]). These complexes promote transcriptional elongation via phosphorylation of the C-terminal domain (CTD) of the large subunit of RNA polymerase II (RNAPII) and subunits of the negative elongation factors DSIF (DRB Sensitivity Inducing Factor) and NELF (Negative Elongation factor)[3]. CDK9 preferentially triggers phosphorylation of Pro-directed Ser/Thr sites [4]. Phosphorylation of RNAPII by CDK9 has been reported to occur preferentially on Ser-2 rather than Ser-5 on the seven-amino acid repeats that form the CTD [5]. However, we have previously shown that in at least two cell lines inhibition of CDK9 activity with flavopiridol (FVP) or a dominant negative mutant of CDK9 results in inhibition of both Ser-2 and Ser-5 phosphorylation on the CTD of RNAPII [6,7]. Many genes are regulated at the elongation rather than the initiation phase of transcription and P-TEFb is recruited by numerous transcription factors near the transcription start sites of many of these promoters. CDK9 inhibition has been studied as a potential therapeutic target for multiple pathologies, including, AIDS, cardiac hypertrophy, inflammation and cancer ([8-14]). We and others have determined the effect of inhibiting CDK9 activity in global gene expression by different means including pharmacological inhibitors [6,7,15,16], a dominant negative mutant of CDK9 [6] and RNA interference targeted to various P-TEFb subunits [17-19] using a variety of cells. Effects on gene expression have been clearly seen in all instances, but it is unclear if the differential patterns of gene expression reflect cell type and/or signal specific differences, or alternatively the differences are due to the kinetics of CDK9 inhibition, potency or the potential off target effects specific to each methodology. Off target effects may include lack of selectivity in pharmacological approaches, anti-viral effects due to viral transduction in dominant negative and RNA interference approaches, as well as squelching effects in both dominant negative and RNA interference approaches.

In this study we sought to compare global changes in gene expression resulting from inhibition of CDK9 activity by different means in hTERT immortalized normal human fibroblasts and primary astrocytes, as well as compare them to previous data obtained using T98G glioblastoma cells [6].

### Results and discussion

We had previously reported differential effects on gene expression in human glioblastoma T98G cells when CDK9 activity was either inhibited pharmacologically with FVP or with a dominant negative form of CDK9 (dnCDK9) expressed from an adenoviral vector [6]. Changes in mRNA expression were determined via Affymetrix microarray analysis. The effects of FVP in mRNA expression were consistent with global inhibition of transcription, whereas inhibition of CDK9 activity with dnCDK9 had more restricted and distinctive effects, with the expression of slightly more genes being upregulated that downregulated [6]. These data suggested that the broader and more potent inhibitory effects of FVP in transcription could be due to the inhibition of other CTD kinases. This is conceivable, because although FVP IC_50_ for CDK9 is 4 fold lower than for other tested CDKs, the IC_50_ for several other CDKs [13,20], including some with the ability to phosphorylate the CTD of RNAPII has not been reported. However, it must be considered that the differences in the mechanisms of CDK9 inhibition, as well as the inhibition kinetics and potency may explain at least some of the distinctive gene expression effects. While FVP inhibits CDK9 activity very potently and rapidly, inhibition with dnCDK9 is much slower, depending on expression of the dominant negative mutant and replacement of endogenous CDK9 from T-type cyclin complexes within the cell. Moreover, since transduction of cells with adenoviruses has an effect on gene expression [21], we could not rule out that vector effects contributed to the differences in gene expression between FVP and dnCDK9.

#### Effects of dnCDK9 and FVP in adenovirally transduced human BJ-TERT fibroblasts

**Figure 1:**
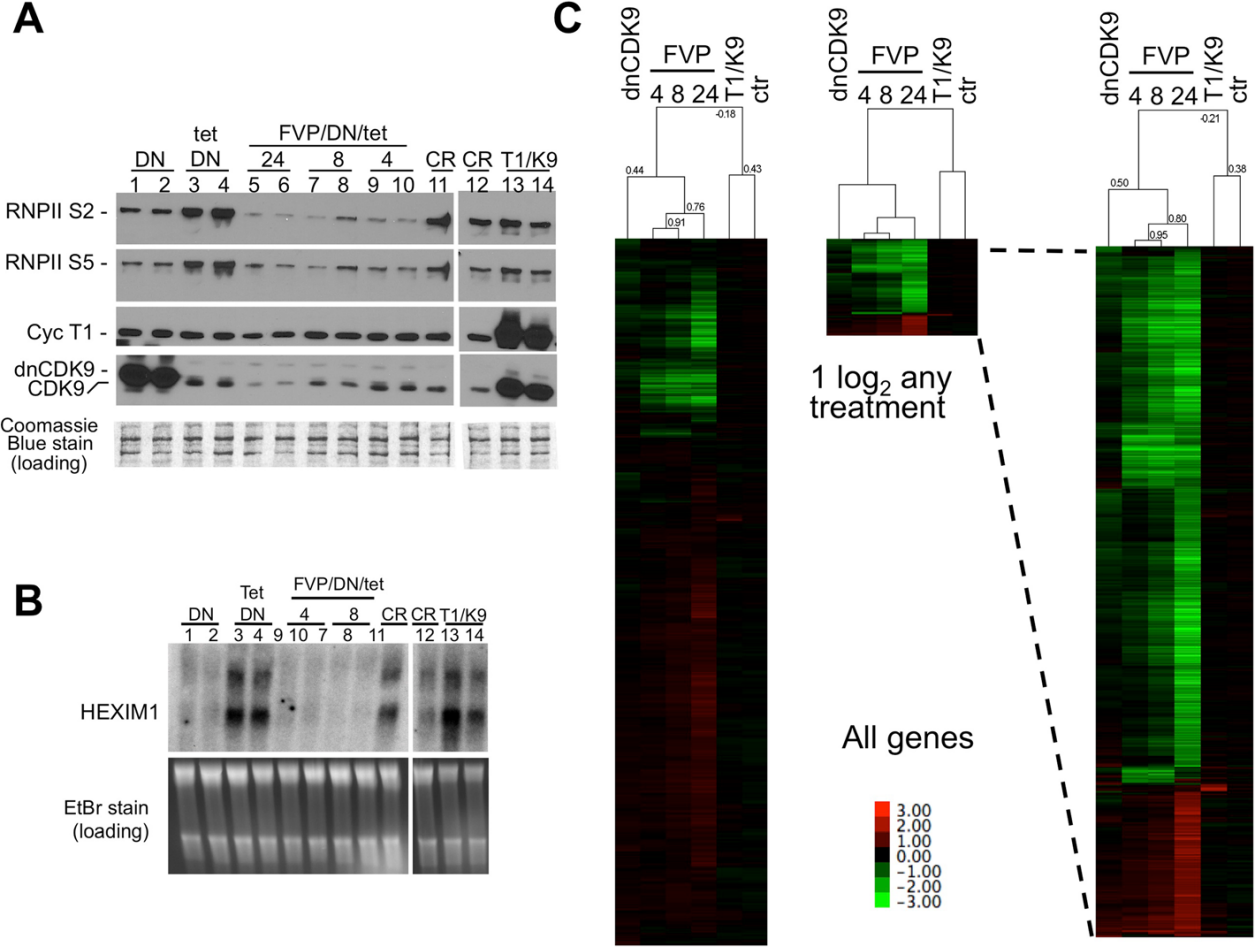
**Effects of CDK9 inhibition on the phosphorylation of the CTD of RNAPII and the expression of genes in hTERT-immortalized normal human fibroblasts.** CDK9 activity was inhibited in BJ-TERT fibroblasts via adenoviral mediated transduction of a tetracycline-repressible (tet) dominant negative CDK9 mutant (DN) or by pharmacological treatment with 300 nM flavopiridol (FVP). No functionally significant DN expression occurs in the presence of tetracycline (lanes 3 to 10). FVP treated cells were also previously transduced with the same adenoviruses and cultured in the presence of tetracycline (no DN effect) to normalize for viral effects as described in the text. All treatments were done in triplicate and duplicate samples are shown in A and B. The other replicate performed separately but under the same conditions exhibited virtually the same effects (not shown). Ectopic expression of dnCDK9 or treatment with FVP inhibit both RNAPII Ser-2 and Ser-5 phosphorylation. **(A)** and the expression of *HEXIM1* transcripts. **(B)** as determined by western and northern blot analysis, respectively. Coomassie Blue (A) and EtBr (B) stains are shown for loading controls. **(C)** Global gene expression effects of ectopic expression of dnCDK9 or FVP treatment in BJ-TERT fibroblasts. Normalized Affymetrix microarray data (log_2_ ratios, see the text in the results section) for all transcripts of triplicate samples were analyzed by correlation uncentered, average linkage, hierarchical clustering (left heat map). Transcripts whose levels changed +/- 1 log_2_ in any treatment were reclustered (middle heat map) and the heat map was magnified for clarity (right heat map). See the text in the results section for details. Correlations are shown on the top of the arrays. A heat map legend is shown.

To extend this study, we selected a non-transformed cell line of normal human fibroblasts immortalized with human telomerase (hTERT), designated BJ-TERT fibroblasts. In our hands these cells have proliferation properties similar to those exhibited by parental non-immortal BJ fibroblasts [22,23]. To eliminate the effects of transduction as a cause of differential effects in gene expression, all cells were transduced identically using the inducible Adeno-X™ Tet-Off system from Clontech carrying a dnCDK9 transgene under a tetracycline repressible promoter. BJ-TERT cells were transduced with the same sets of adenoviruses (Ad-X-dnCDK9 and Ad-Tet-off). Tetracycline was added to the media of the control cells (tet-DN), as well as cells to be treated with FVP (FVP/DN/tet) to prevent expression of dnCDK9, while cells selected to express dnCDK9 were allowed to grow in the absence of tetracycline (DN, see methods). Under these experimental conditions, the baseline gene expression was the same in all samples prior to inhibition of CDK9 by different means. Forty-eight hours post-transduction, we analyzed the effects of CDK9 inhibition on phosphorylation of RNAPll on serine position 2 (Ser-2) and 5 (Ser-5) using specific antibodies (Figure 1A). CDK9 is thought to phosphorylate Ser-2 during transcription elongation, while CDK7 phosphorylates Ser-5 at initiation [5]. However, and consistent with our previous observations using T98G cells [6], both Ser-2 and Ser-5 were found to be clearly dephosphorylated in BJ-TERT cells expressing dnCDK9 or treated with FVP (Figure 1A, compare lanes 1–2 and 5–10 to lanes 3–4). BJ-TERT fibroblasts transduced in parallel in the presence of tetracycline were treated with 300 nM FVP for the indicated last 4, 8 and 24 h prior to the 48 h collection time (under these conditions cells remain attached to the tissue culture dish and show no signs of overt cell death). FVP inhibited Ser-2 and Ser-5 phosphorylation at all time points more efficiently than dnCDK9. Consistent with its global inhibitory effect on transcription, the levels of endogenous CDK9 and, less prominently, cyclin T1 (24 h time point) were also downregulated by the FVP treatment (Figure 1A) and total protein concentration significantly decreased by the 24 h time point (data not shown). We also verified the inhibitory effects of dnCDK9 and FVP on the expression of *HEXIM1*, a gene known to be modulated by P-TEFb, which is very sensitive to CDK9 inhibition [6,24]. As shown in Figure 1B, the *HEXIM1* mRNA levels are downregulated by both dnCDK9 and FVP. The effects of overexpressing cyclin T1 and CDK9 were also monitored. No major effects were noticed in the phosphorylation of the CTD of RNAPII or the expression of *HEXIM1* mRNA, as compared to control cells infected with Ad-Cre, expressing the Cre recombinase.

We next performed a global gene expression profiling of the effects of inhibiting CDK9 in BJ-TERT fibroblasts by using Affymetrix Human Gene 1.0 ST DNA arrays and total RNAs from the samples described above in triplicate. These arrays contain probes representing 28,869 different genes. RNAs were labeled, hybridized to microarrays and scanned as described in [6]. Raw transcript intensity data were normalized and the expression value log_2_ ratio for each gene was computed between its treatment and corresponding control sample (tet/DN was the control for DN and the FVP/DN/Tet treatments; Ad-Cre (CR) was the control for Ad-T1/K9 (T1/K9); and ctr is the ratio of the two control treatments tet/DN vs. Ad-Cre (CR)). Principal component analysis demonstrates that all biological replicates cluster together (Additional file 1: Figure S1). We represented the average gene expression value between replicates as the average log_2_ ratio. Next, hierarchical cluster analysis was performed with the log_2_ ratios and visualized using by Java Tree View (Figure 1C). Gene array ratios for the three FVP treatments clustered together (correlation: 0.76-0.91), but exhibited much lower correlation with the dnCDK9 gene array ratios (correlation: 0.44). If only the probe sets that change two fold or more (log_2_ ratios ≤ -1 or ≥1) with at least one treatment are considered, this correlation increases slightly (0.50)(Figure 1C, right clusters). Interestingly, ectopic expression of P-TEFb subunits (cyclin T1 and CDK9) resulted in the marked upregulation of a small subset of genes, with mostly no effect on genes dramatically upregulated/downregulated by FVP or dnCDK9. Of note, the small subset of genes upregulated were highly enriched with interferon response genes, which could indicate that in virally transduced cells P-TEFb activity is limiting (Additional file 2: Figure S2). As expected, very little difference was observed in the expression profile of both control cell treatments: cells transduced with Ad-X-dnCDK9/Ad-Tet in the presence of tetracycline versus Ad-Cre (Figure 1C, ctr (tet/DN vs. CR), right array in all clusters).

**Figure 2:**
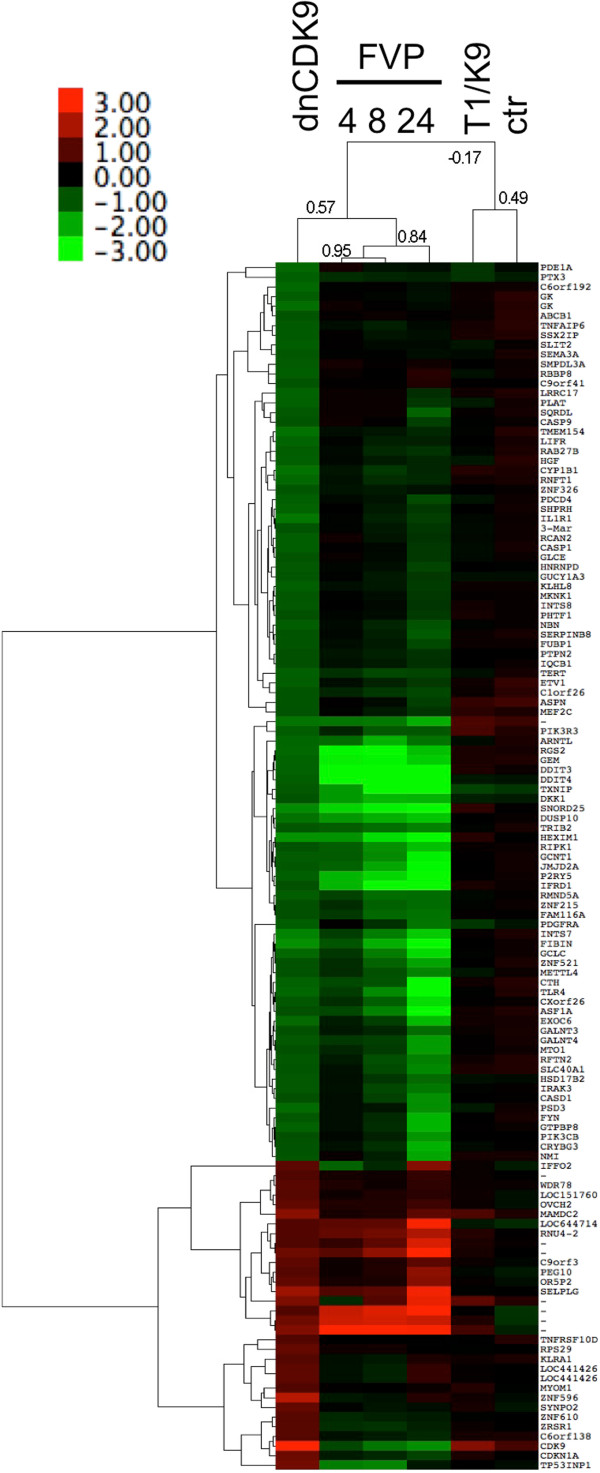
**Effects of FVP treatment on genes modulated by dnCDK9 in hTERT-immortalized normal human fibroblasts.** Affymetrix microarray expression ratios for transcripts changing +/-1 log_2_ with ectopic expression of dnCDK9 were clustered with the corresponding values of the FVP treatments using hierarchical clustering analysis (correlation uncentered, average linkage). Correlations among treatments are shown on the top dendogram. Gene clusters are shown on the left of the heat map. A heat map legend is shown.

Analysis of the probe sets that change approximately two fold or more (log_2_ ratios ≤ -1 or ≥1) upon dnCDK9 expression showed that 93 probe sets were downregulated and 32 were upregulated (Figure 2). The highest downregulation corresponded to *HEXIM1* (log_2_ = -1.72043), confirming the sensitivity of *HEXIM1* to CDK9 inhibition. As expected, FVP also potently inhibited *HEXIM1* expression (log_2_ = -1.72494 to -3.22926). The highest probe set intensity in the dnCDK9 array was CDK9, an obvious expected result due to the ectopic expression of dnCDK9, as the probe set does not discriminate between dnCDK9 and endogenous CDK9. Of note, FVP inhibited the expression of endogenous CDK9. The correlation between the FVP arrays was 0.95 for the 4 and 8 h treatments and 0.84 with the 24 h treatment. It was also clear that the increase/decrease in gene expression with FVP was time dependent. The correlation between the FVP and dnCDK9 arrays was 0.57, noticeably higher than we had seen in our previous study using T98G cells [6]. The lowest log_2_ ratio was -1.7 and the highest was 2.2, while the expression change range for FVP at 24 h was much higher (-5.4 to 4.2). Therefore, one cannot conclude that the effects of dnCDK9 are more or less potent than any of the FVP treatments for this set of genes but rather different.

#### Global effect on gene expression with loss of CDK9 function via siRNA

**Figure 3:**
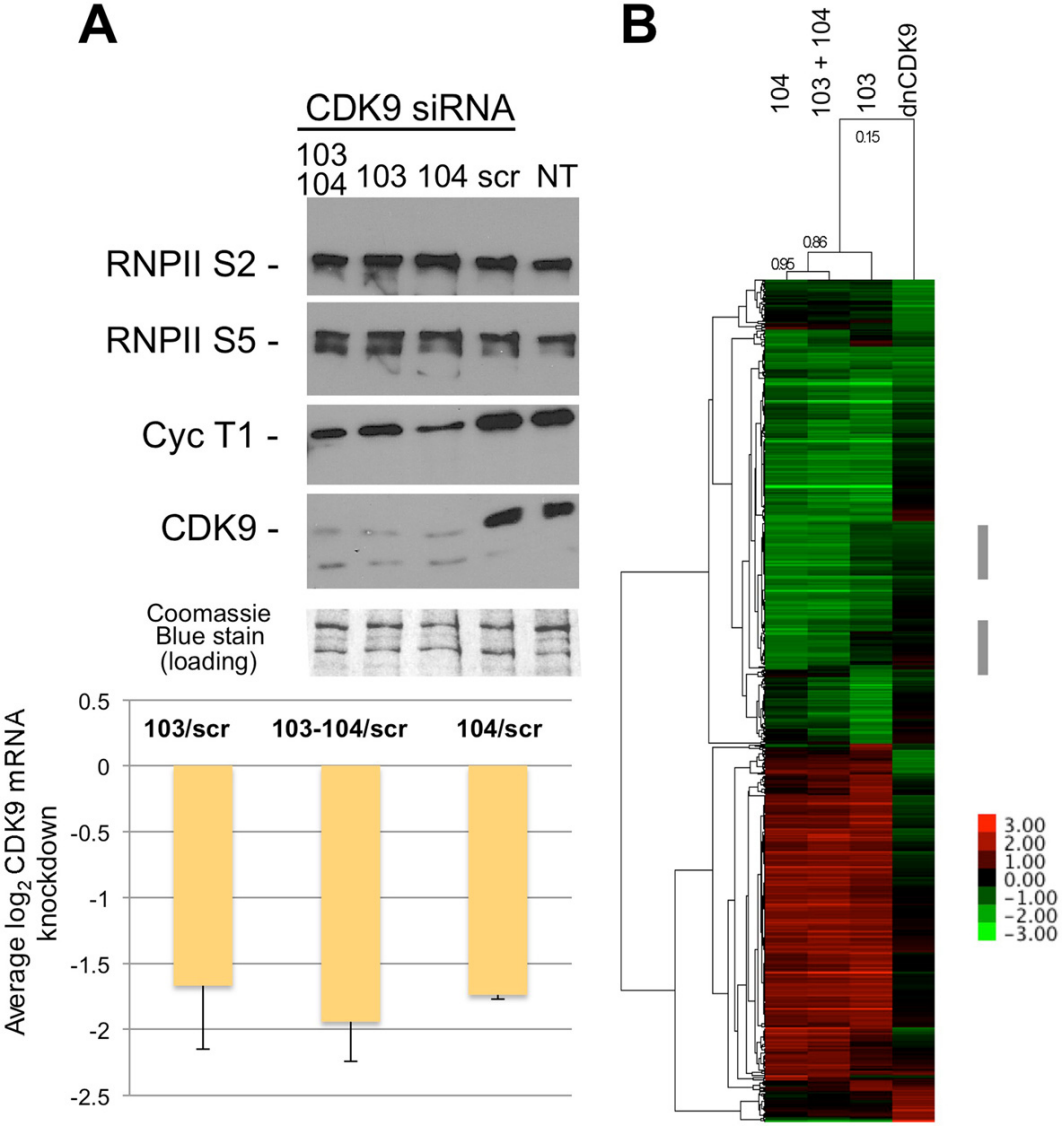
**Very limited correlation among genes modulated by siCDK9 and dnCDK9 in hTERT immortalized NHF. (A)** Knockdown of CDK9 results in reduced cyclin T1 expression, but does not affect RNAPII phosphorylation noticeably. The Coomassie Blue stained membrane is shown as loading control. *NT* indicates untransfected cells and *scr* indicates scramble siRNA.** (B)** log_2_ expression ratios for transcripts which levels changed a +/-1 log_2_ ratio or more with siCDK9 (103 and/or 104 vs. scr) or dnCDK9 (DN vs. tet/DN) expression were hierarchically clustered using Cluster 3.0 (correlation uncentered, average linkage)(~650 transcripts are shown). The correlation among arrays is show on top. A heat map legend is shown.

The observation that many of the genes more sensitive to dnCDK9 or FVP treatment are different suggest that one or both methods lack selectivity or have off target effects. For instance, FVP may inhibit other CDKs with roles in transcription in addition to CDK9, even at the concentrations used in this study. One could also envision dnCDK9 having additional effects unrelated to CDK9 inhibition resulting from squelching P-TEFb partners. Alternatively, the differences could be explained by the difference in kinetics of inhibition of FVP compared to dnCDK9. FVP acts very rapidly and potently, while inhibition by dnCDK9 occurs slowly, depending on expression and replacement of active complexes by inactive ones. In view of these results, we decided to broaden our study by reducing CDK9 expression via siRNA. This is a priori a very specific and selective method, as no other RNA sequences are targeted and, thus, other RNAPII CDKs should not be directly affected. BJ-TERT fibroblasts were transfected twice within 24 h with two independent siRNAs (103 and 104) and collected 72 h after the first transfection. We next determined the effect of each siRNA and their combination on CDK9 levels. Both 103 and 104 siRNAs efficiently knocked-down CDK9 mRNA and protein (Figure 3A), with little additional downregulation when both siRNAs were used in combination. As expected scramble siRNAs had no effect on CDK9 levels. However, in contrast to FVP and dnCDK9 treatments (Figure 1A), phosphorylation of Ser-2 and Ser-5 of the CTD of RNAPII were not visibly affected by either siCDK9 or their combination (Figure 3A). The effects of knocking-down CDK9 in global gene expression in BJ-TERT fibroblasts were determined using Affymetrix Human Gene 1.0 ST DNA arrays similarly as described for dnCDK9 and FVP treated cells. Cells were transfected in triplicate with each siCDK9 or combination or in duplicate with scramble siRNA. Cells were collected 72 h later to generate RNA probes (see Methods). About 650 probe sets exhibited changes in gene expression equal or larger than two fold (log_2_ ratios ≤ -1 or ≥1). A cluster analysis of genes that changed two fold or more with siCDK9 or dnCDK9 is shown in Figure 3B. The correlation between siCDK9 treatments was high (0.86-0.95). Both the 103 and 104 siRNAs exhibit some unique set of genes that were more potently affected, with the combination appearing to be additive for those genes. However, for genes that were commonly downregulated or upregulated by both siRNA separately, the combination mostly lacked additive effects (Figure 3B, side grey bars). This may reflect that both siRNAs knocked-down CDK9 to similar levels even when used in combination, as the total siRNA concentration was the same in all transfections. Surprisingly clustering analysis also revealed major differences between the effect of the siRNAs and dnCDK9 (correlation 0.15). For instance a large set of genes upregulated by dnCDK9 are not affected by either siRNA (bottom large cluster in the array). Similarly, the top of the array shows several genes potently downregulated by dnCDK9 that show minimal downregulation by siRNA. In addition, most of the genes upregulated by either 103, 104 or the combination of both are either not affected or downregulated by dnCDK9. While there is more agreement within the genes downregulated by the siRNAs, the overall comparison exhibits little similarity.

**Figure 4:**
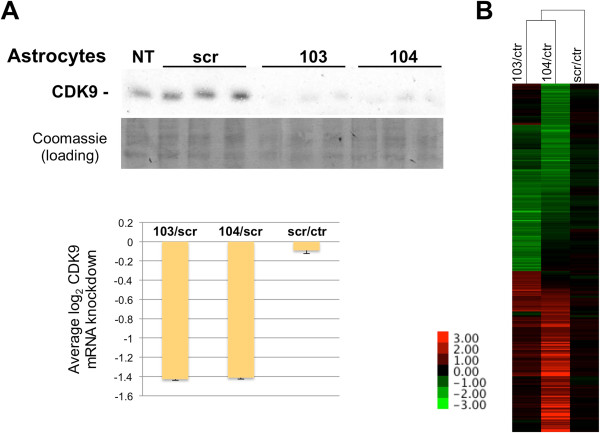
**Effect of CDK9 knockdown on gene expression in primary astrocytes. (A)** Knockdown of CDK9 results in markedly reduced CDK9 expression (siRNA experiments were done in triplicate as shown). A representative portion of the Coomassie Blue stained PDVF membrane is shown as loading control. *NT* indicates untransfected cells and *scr* indicates scramble siRNA. **(B)** log_2_ expression ratios for transcripts which levels changed a +/-log_2_ ratio or more with siCDK9 after elimination of genes which expression was altered nonspecifically by transfection of scr siRNA (see text) were clustered (correlation uncentered, average linkage). Correlation distances are shown on the top dendogram. A heat map legend is shown.

The effect of these siRNAs were also determined in primary human astrocytes. Cells were transfected in duplicate with siRNAs targeting CDK9 (103 and 104), scramble or mock transfected and cells were harvested 72 h later. As with BJ-TERT fibroblasts, 103 and 104, but not scramble knocked down CDK9 mRNA and protein (Figure 4A). RNA was prepared from transfected and untransfected cells for microarray analysis and data were analyzed as above. Untreated cells were analyzed in this experiment to determine whether any of the effects observed in siRNA transfected cells could be due to experimental manipulation. We observed that transfection had significant effects in the expression of interferon response genes as well as other genes. Transfection with one of the siRNAs directed to CDK9 (104) had additional effects on the expression of these genes, which were dramatically upregulated, but were not upregulated with the other siRNA directed to CDK9 (103). Elimination of those genes in clustering analysis allows focusing in the potentially regulated genes, defined as those affected by transfection with both 103 and 104, but not scr, as compared to untransfected cells (Figure 4B).

**Figure 5:**
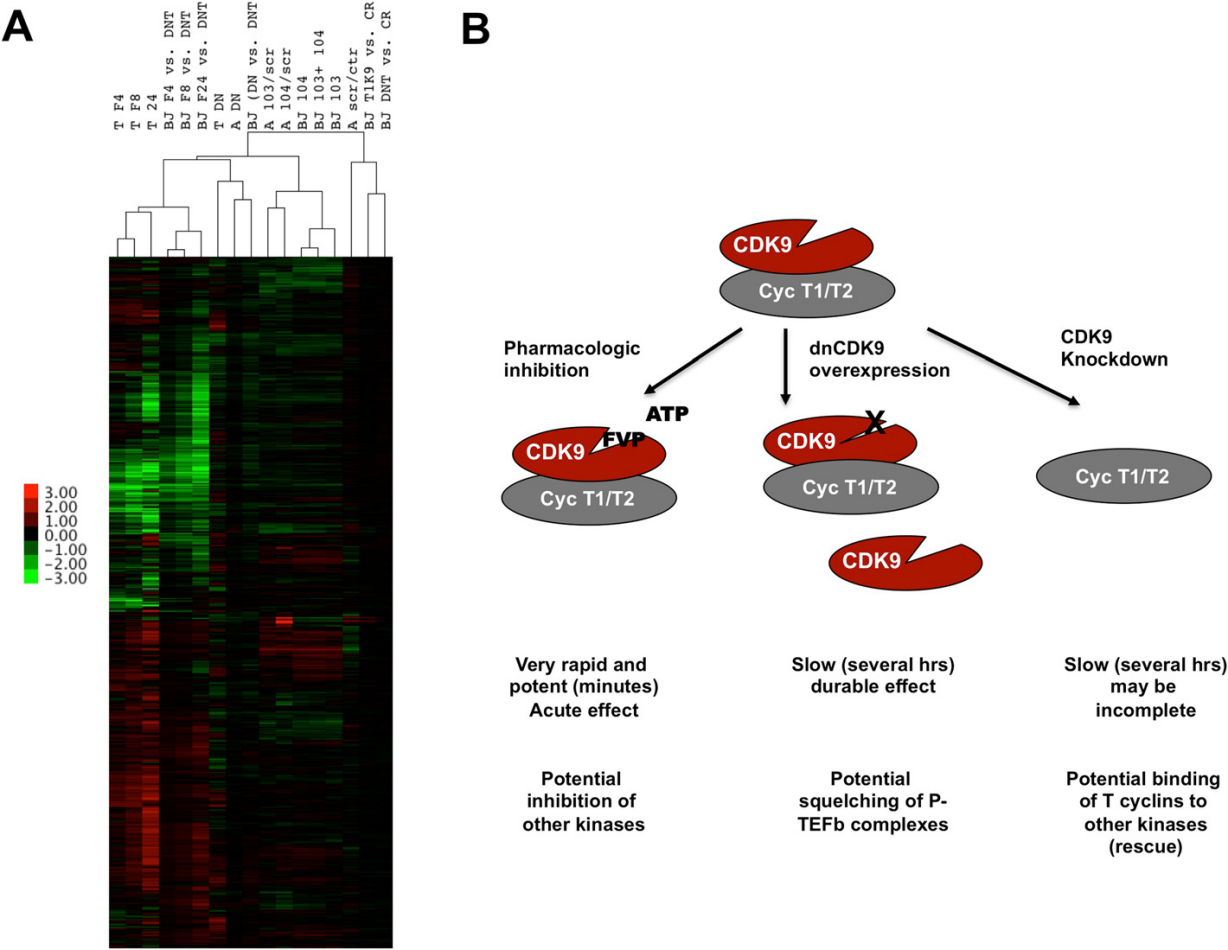
**Hierarchical Cluster analysis of al treatments. (A)** Genes with +/-1 log_2_ ratios for treatments described in this study and [6] were clustered as described in the text and previous figures. Cell treatments are indicated on top. *T* indicates T98G cells, *A* indicates astrocytes, *BJ* indicates BJ-TERT fibroblasts. FVP treatments are indicated by *F4, F8 or F24.* Correlation distances are shown on the top of the arrays. A heat map legend is shown. **(B)** Schematic representation of the three strategies used to inhibit CDK9 activity, their mechanism, speed and potential off target effects.

Finally, we combined all microarray data from this study and our previously published data using T98G cells [6] to perform hierarchical cluster analysis. Interestingly, this analysis shows that gene arrays cluster by treatment rather than cell type or transformation status (Figure 5A). Specifically, the FVP treatments of T98G and BJ-TERT fibroblasts clustered together, as did siRNA transfected cells or cells expressing dnCDK9. These data show that the type of treatment induces clear signatures shared in across cell types that are more prominent than cell type specific signatures that could result from inhibition of CDK9 by any methodology (Figure 5B).

In summary, it is clear that the strategy selected to inhibit CDK9 activity in cells contributes to the changes in gene expression observed and, thus, caution needs to be observed in the interpretation of data, especially when comparing results obtained using different strategies (Figure 5B). Because pharmacologic inhibitors such as FVP act rapidly (within minutes of treatment), the observed effects in gene expression are acute and more likely to be direct if the pharmacologic inhibitor is selective in cells. In our hands, FVP inhibits phosphorylation of RNAPII on both Ser-2 and Ser-5 (Figure 1), even when used for shorter periods of time [7]. Also, using lower concentrations of FVP in cells did not result in significantly greater selectivity [7]. So, it cannot be ruled out that the potency of FVP is in part due to inhibition of other CTD kinases with roles in transcriptional elongation and responsible for Ser-5 phosphorylation. On the other hand, it is also conceivable that CDK9 phosphorylates both Ser-2 and Ser-5 in cells, and this is consistent with the results obtained with dnCDK9, which inhibits phosphorylation of both. Surprisingly, we did not observe major changes in the phosphorylation state of both Ser-2 and Ser-5 upon CDK9 knockdown, which might suggest that in the absence of CDK9 other CDKs may act redundantly to maintain overall CTD phosphorylation in cells. It is anticipated that development of more selective pharmacologic inhibitors of CDK9 will help resolve the open questions in the future.

These observations are significant because CDK9 is being evaluated as a therapeutic target for a number of pathologies and the efficacy of treatments targeting CDK9 would be predicted to be highly variable depending on the approach used to inhibit CDK9 activity.

### Methods

#### Cell culture

BJ-TERT fibroblasts are normal human BJ fibroblasts immortalized with human Telomerase Reserve Transcriptase (hTERT) [25]. BJ-TERT fibroblasts were grown in Dulbecco’s Modification of Eagle’s Medium (DMEM) (Cellgro) supplemented with 10% Fetal Bovine Serum (FBS) (Sigma) and antibiotics at 37°C in 5% CO_2_. Normal human astrocytes (NHA) were purchased from Lonza and grown in astrocyte basal media (ABM™) plus growth factors (Lonza) at 37°C and 5% CO_2_ following manufacturer’s directions. NHA have a limited life-span, but can be expanded up to five passages.

#### Cell treatments

CDK9 was pharmacologically inhibited by adding Flavopiridol (FVP) to the medium to a final concentration of 300 nM. FVP was dissolved in DMSO to generate a 10 mM stock and then diluted in PBS to 100 μM and added to the medium. Control cells were treated with the same concentration of DMSO (Dimethyl sulfoxide) dissolved in PBS. FVP treated cells were preinfected with Ad-T-dnCDK9 plus Adeno-X™ Tet-Off™ adenoviruses in the presence of tetracycline (tet/DN) to eliminate viral transduction as a variable in the comparison to dnCDK9 expressing cells (transduced with Ad-T-dnCDK9 plus Adeno-X™ Tet-Off™ in the absence of tetracycline (DN)). BJ-TERT fibroblasts were infected with Ad-T-dnCDK9 plus Adeno-X™ Tet-Off™ adenoviruses (dn) in the presence or absence of tetracycline (tet) essentially as previously described [6] and then treated with FVP for 8, 4 and 24 h. Double stranded siRNAs were obtained from Ambion and had the following sense sequences: 103, 5′-GGUGCUGAUGGAAAACGAGtt-3′ and 104, 5′-GGAGAAUUUUACUGUGUUUtg-3′. The scrambled siRNA, Scr, 5′- AAUUCUCCGAACGUGUCACGU-3′ was purchased from Qiagen. BJ-TERT fibroblasts (0.5 × 10^6^) were seeded in 10 cm plates 14 h prior to transfection. SiRNAs were transfected with Dharmafect using manufacturer-established protocols. The Dharmafect/siRNA/antibiotic free medium mix was incubated for 20 min at room temperature and added to cells re-fed with fresh antibiotic free medium. NHA (1 × 10^6^) were transfected with 50 nM siRNA in the presence of 15 μL Lipofectamine RNAimax (Life Technologies) in serum free media (Optimem, Life Technologies). Media was changed 6 hours later and cells were collected to process for analysis 72 hours post-transfection.

#### Protein and RNA analyses

Cells were lysed with ice-cold lysis buffer (50 mM Tris–HCl (pH 7.4), 5 mM EDTA, 250 mM NaCl, 50 mM NaF, 0.1 mM Na_3_VO_4_, 0.1% Triton X-100, 2 mM PMSF, 4 μg/ml aprotinin, 10 μg/ml leupeptin, and 40 μg/ml pepstanin). Protein concentration was determined using the BioRad protein reagent and BSA to generate a standard curve, with all protein sample replicates reading in the linear range. Proteins were resolved by 8% polyacrylamide/SDS gel electrophoresis, and transferred to a polyvinylidene difluoride (PVDF) membrane in 10 mM CAPS/10% methanol buffer (pH 11). ECL Western blot analyses were performed as described previously [6] using the following antibodies: Anti-RNAPII (A300-653A), anti-Ser-2 (A300-654A), and anti-Ser-5 (A300-655A) rabbit polyclonal antibodies were obtained from Bethyl.

Total RNA was isolated from cells using QIAshredder and Rneasy Mini Kit from Qiagen. Northern Blots were performed as previously described [23].

#### Affymetrix microarray hybridization, quantitation and hierarchical cluster analysis, and visualization

Global analysis of gene expression was performed using Affymetrix Human Gene 1.0 ST microarrays, which contains probe for 28,869 non-redundant transcripts. RNA was labeled and hybridized to microarrays at the University of Pennsylvania Microarray Facility (U. Penn MF) following manufacturer directions. Microarray intensity values were normalized using the Affymetrix Expression Console Software. The average of log_2_ ratios was calculated to represent average gene expression levels among replicate arrays. Hierarchical cluster analysis was performed using Cluster version 3.0 software with average linkage [26] and visualized with Java TreeView, version 1.0.13 as in [6].

### Availability of supporting data

Microarray expression data will be deposited in the GEO database upon manuscript acceptance.

## Abbreviations

Ad: Adenovirus; CDK9: Cyclin Dependent Kinase 9; CTD: C-terminal domain; DMSO: Dimethyl sulfoxide; DN: Dominant negative; DSIF: DRB Sensitivity Inducing Factor; hTERT: Human Telomerase Reverse Transcriptase; NELF: Negative Elongation factor; P-TEFb: Positive Transcription Elongation Factor b; RNAPII: RNA polymerase II.

## Competing interests

The authors’ declare that they have no competing interests.

## Authors’ contributions

JG and XG. Performed cell treatments and microarray experiments, analyzed the data and wrote the manuscript. Both authors read and approved the final manuscript.

## Supplementary Material

Additional file 1: Figure S1 Principal component analysis shows that all biological replicates cluster together. Principal component analysis was performed with normalized data for all treatments described in Figure 1. Biological replicates of the same treatment are colored, as shown in the color legend on the right. Click here for file

Additional file 2: Figure S2 Ectopic expression of P-TEFb subunits (cyclin T1 and CDK9) resulted in the marked upregulation of a small subset of genes highly enriched with interferon response genes. Ingenuity Pathway Analysis (IPA) was performed with the set of genes upregulated in BJ-TERT cells ectopically expressing cyclin T1 and CDK9 as compared to cells transduced with the Ad-Cre control virus. Genes involved in cell-immediate immune response and humoral response are highly enriched (A). IPA Network analysis of the upregulated genes in BJ-Tert fibroblasts ectopically expressing Cyclin T1 and CDK9 identified the interferon network, which shows upregulation of multiple interferon response genes. Click here for file
