# Supplementary figures and images for: CDK9 inhibition strategy defines distinct sets of target genes
Source: BMC Res Notes. 2014 May 16;7:301. doi: 10.1186/1756-0500-7-301 (PMC4045923; doi:10.1186/1756-0500-7-301)

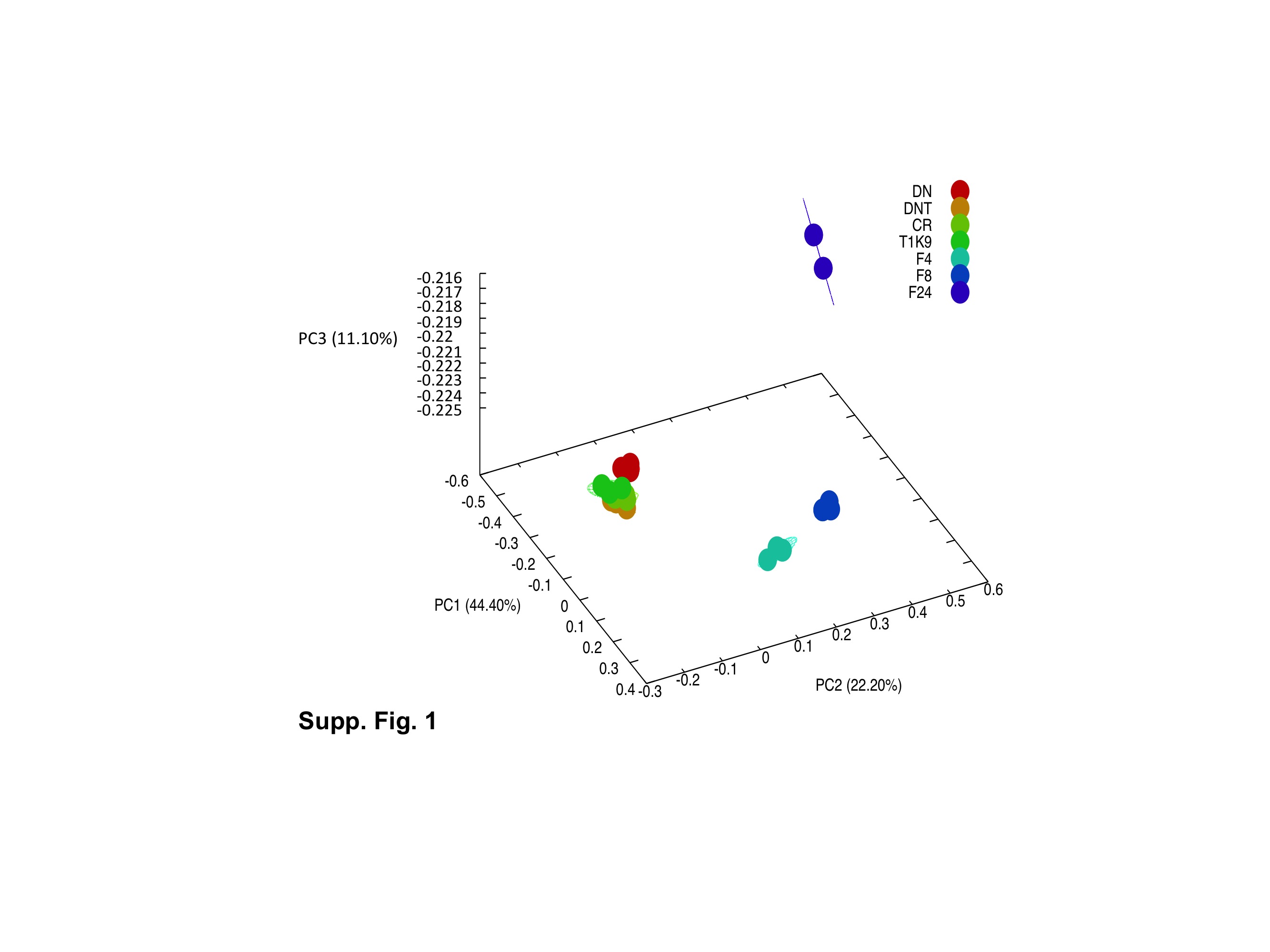

Supplement: Additional file 1: Figure S1 — Principal component analysis shows that all biological replicates cluster together. Principal component analysis was performed with normalized data for all treatments described in Figure 1. Biological replicates of the same treatment are colored, as shown in the color legend on the right. [file 1756-0500-7-301-S1.jpeg]

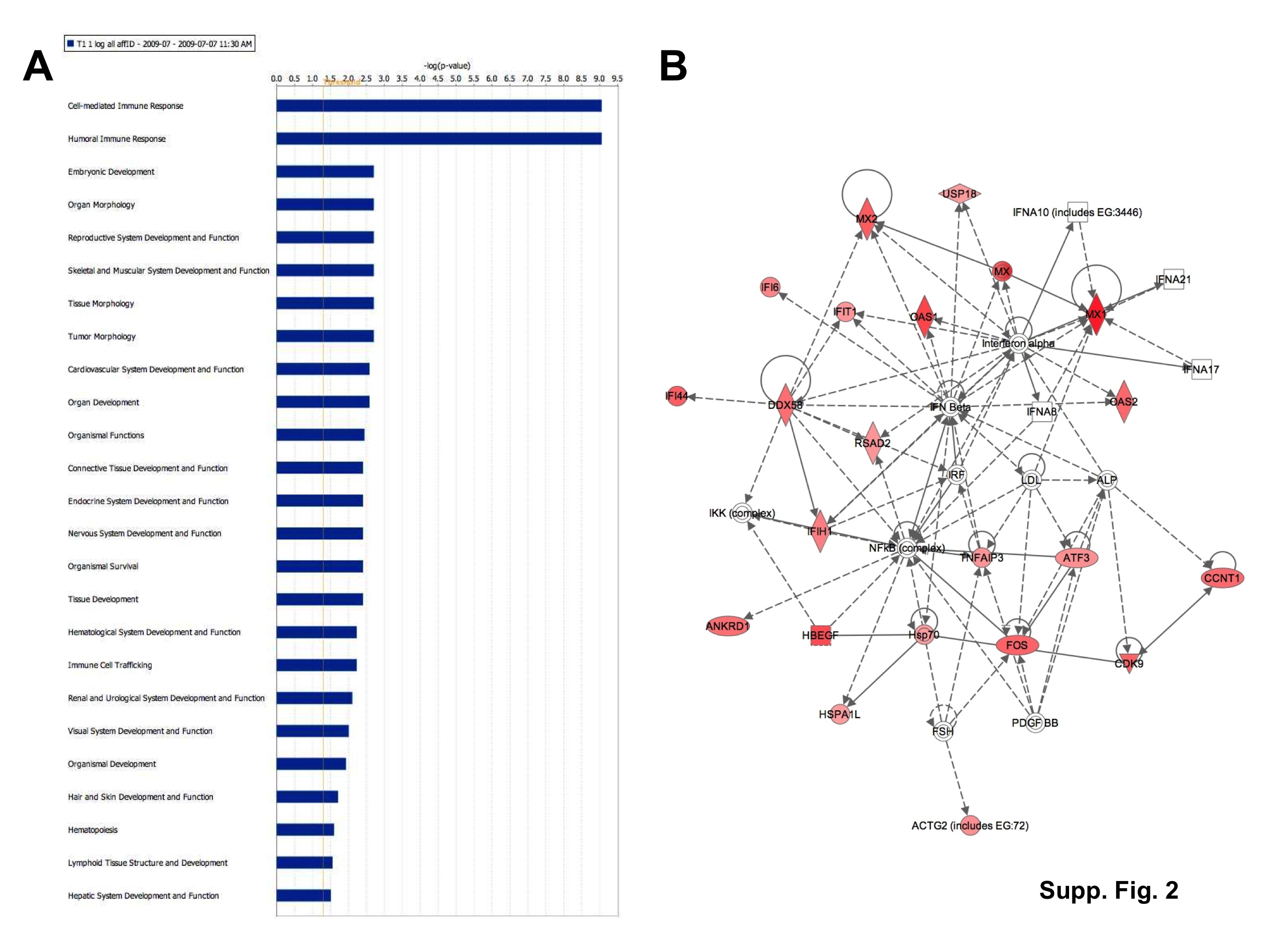

Supplement: Additional file 2: Figure S2 — Ectopic expression of P-TEFb subunits (cyclin T1 and CDK9) resulted in the marked upregulation of a small subset of genes highly enriched with interferon response genes. Ingenuity Pathway Analysis (IPA) was performed with the set of genes upregulated in BJ-TERT cells ectopically expressing cyclin T1 and CDK9 as compared to cells transduced with the Ad-Cre control virus. Genes involved in cell-immediate immune response and humoral response are highly enriched (A). IPA Network analysis of the upregulated genes in BJ-Tert fibroblasts ectopically expressing Cyclin T1 and CDK9 identified the interferon network, which shows upregulation of multiple interferon response genes. [file 1756-0500-7-301-S2.jpeg]
